# Sheep Milk Symbiotic Ice Cream: Effect of Inulin and Apple Fiber on the Survival of Five Probiotic Bacterial Strains during Simulated In Vitro Digestion Conditions

**DOI:** 10.3390/nu14214454

**Published:** 2022-10-23

**Authors:** Magdalena Kowalczyk, Agata Znamirowska-Piotrowska, Magdalena Buniowska-Olejnik, Małgorzata Pawlos

**Affiliations:** Department of Dairy Technology, Institute of Food Technology and Nutrition, College of Natural Sciences, University of Rzeszow, Ćwiklińskiej 2D, 35-601 Rzeszów, Poland

**Keywords:** ice cream, probiotic, prebiotic, bacterial survival rate, in vitro

## Abstract

We conducted a study to determine the survival of bacterial cells under in vitro digestion. For this purpose, ice cream mixes were prepared: control, with 4% inulin, 2.5% inulin and 1.5% apple fiber and 4% apple fiber. Each inoculum (pH = 4.60 ± 0.05), containing 9 log cfu g^−1^ bacteria, at 5% (*w/w*) was added to the ice cream mixes (*Lacticaseibacillus* *casei* 431, *Lactobacillus acidophilus* LA-5, *Lacticaseibacillus paracasei* L-26, *Lacticaseibacillus* *rhamnosus*, *Bifidobacterium animalis* ssp. *lactis* BB-12) and fermentation was carried out to pH 4.60 ± 0.05. The in vitro digestion method simulated the stages of digestion that occur in the mouth, stomach and small intestine under optimal controlled conditions (pH value, time and temperature). At each stage of digestion, the survival rate of probiotic bacteria was determined using the plate-deep method. As expected, in the oral stage, there was no significant reduction in the viability of the probiotic bacteria in any ice cream group compared to their content before digestion. In the stomach stage, *Bifidobacterium animalis* ssp. *lactis* BB-12 strain had the highest viable counts (8.48 log cfu g^−1^) among the control samples. Furthermore, a 4% addition of inulin to ice cream with *Bifidobacterium* BB-12 increased gastric juice tolerance and limited strain reduction by only 16.7% compared to the number of bacterial cells before digestion. Regarding ice cream samples with *Bifidobacterium* BB-12, replacing part of the inulin with apple fiber resulted in increased survival at the stomach stage and a low reduction in the bacterial population of only 15.6% compared to samples before digestion. At the stomach stage, the positive effect of the addition of inulin and apple fiber was also demonstrated for ice cream samples with *Lacticaseibacillus* *casei* 431 (9.47 log cfu g^−1^), *Lactobacillus acidophilus* LA-5 (8.06 log cfu g^−1^) and *Lacticaseibacillus paracasei* L-26 (5.79 log cfu g^−1^). This study showed the highest sensitivity to simulated gastric stress for ice cream samples with *Lacticaseibacillus* *rhamnosus* (4.54 log cfu g^−1^). Our study confirmed that the 4% addition of inulin to ice cream increases the survival rate of *L. casei* and *Bifidobacterium* BB-12 in simulated intestinal juice with bile by 0.87 and 2.26 log cfu g^−1^, respectively. The highest viable count in the small intestine stage was observed in ice cream with *L. acidophilus*. The addition of inulin increased the survival of *L. rhamnosus* by 10.8% and *Bifidobacterium* BB-12 by about 22% under conditions of simulated in vitro digestion compared to their control samples. The survival rates of *L. casei* and *L. paracasei* were also highly affected by the 4% addition of apple fiber, where the increase under gastrointestinal passage conditions was determined to range from 7.86–11.26% compared to their control counterparts. In comparison, the lowest survival rate was found in the control ice cream with *L. rhamnosus* (47.40%). In our study at the intestinal stage, only five ice cream groups: a sample with 4% inulin and *L. acidophilus*, a control sample with *Bifidobacterium* BB12, a sample with 2.5% inulin and 1.5% apple fiber with *Bifidobacterium* BB12, a control sample with *L. rhamnosus*, a sample with 4% fiber and *L. rhamnosus* reported bacterial cell counts below 6 log cfu g^−1^ but higher than 5 log cfu g^−1^. However, in the remaining ice cream groups, viable counts of bacterial cells ranged from 6.11 to 8.88 log cfu g^−1^, ensuring a therapeutic effect. Studies have clearly indicated that sheep milk ice cream could provide a suitable matrix for the delivery of probiotics and prebiotics and contribute to intestinal homeostasis. The obtained results have an applicative character and may play an essential role in developing new functional sheep milk ice cream.

## 1. Introduction

In the recent decade, it has become common practice to integrate bioactive ingredients into food products to promote health and personal well-being [1]. Ice cream is a product widely known and consumed by all age groups worldwide. Increasing dietary concerns about the impact on health have led to a growing interest in ice cream manufactured with health-promoting ingredients, including prebiotics and probiotic bacteria.

Sheep milk is an excellent raw material for producing probiotic ice cream. Sheep milk differs from cow milk in chemical composition, including higher protein and fat content, and physicochemical properties [2]. High protein content enhances the growth of microorganisms, reducing the fermentation time in the production process. Some researchers have indicated that the fat content of ice cream positively affects the stability of probiotics [3]. According to Ranadheera et al. [2], higher fat and protein levels in sheep’s milk can protect probiotics as they pass through the digestive tract. Moreover, sheep milk contains high levels of calcium, about 200 mg 100 g^−1^ [4]. As an intracellular secondary messenger, calcium ions play a significant role in various cellular pathways, involving almost all cellular reactions required for average cell survival [5]. The addition of Ca^2+^ has been found to increase the yield and biological activity of exopolysaccharides [6,7,8], which may affect the cell survival of probiotic strains in the gastrointestinal tract.

Probiotics are defined as live microorganisms that, when provided in adequate amounts, are beneficial to the host’s health [9,10]. Probiotics are credited with numerous health benefits, including anti-infective, antimutagenic and anticarcinogenic effects, stimulation of the immune system, lowering serum cholesterol levels, improving nutritional value, and relieving symptoms of lactose intolerance [11,12,13,14].

Probiotic bacteria have the ability to spread from the gastrointestinal tract to extraintestinal sites through dendritic cells. These cells can penetrate the epithelium and absorb bacteria directly from the intestinal lumen. After entering dendritic cells or macrophages, probiotic bacteria can be transported to other locations by circulating immune cells in the bloodstream. Bacterial adhesion to the host surface is a crucial aspect of host colonization, as it prevents the mechanical clearance of pathogens [15]. Furthermore, some lactobacilli and bifidobacteria can produce antimicrobial peptides known as bacteriocins, which prevent the proliferation of selected pathogens. Bacteriocins are small cationic molecules composed of about 30–60 amino acids. These molecules act on the cytoplasmic membranes of bacteria and target energized membrane vesicles to disrupt the proton-motive force, thereby inhibiting the growth of many pathogens [16]. Numerous factors regulate the growth and development of probiotic bacteria, primarily optimal temperature, pH value, chemical composition, presence of oxygen and low oxidoreduction potential. Probiotic bacteria have the ability to regulate intracellular pH. This attribute is essential because it allows growth at a pH in the range of 4.5 to 7.0. At a pH value that inhibits the growth of probiotic bacteria, the cytoplasmic membrane is damaged, and it causes bacterial death [17]. Probiotic bacteria are able to produce exopolysaccharides (EPS). These polymers surround probiotic cells, protecting them from harmful conditions and protecting them from cell dehydration, antibiotics, toxic substances, osmotic stress and pathogens [18]. Probiotics have the ability to inhibit intestinal bacterial enzymes that convert procarcinogens into carcinogens [19]. Some strains of probiotic bacteria have been shown to successfully bind to and neutralize aflatoxin B1 (AFB1) in vivo, thereby reducing the bioabsorption of the toxin from the gut [20]. Moreover, probiotics reduce the concentration of fecal enzymes (glycosidase, B-glucuronidase, azoreductase and nitroreductase) and secondary bile salts and reduce the absorption of harmful mutagens that contribute to colon cancer [19]. Probiotic strains of *L. rhamnosus* and *Bifidobacterium lactis* combined with prebiotic inulin enriched with oligofructose provide anticancer effects [21]. *L. acidophilus* and *L. casei* also exhibit immunoenhancing properties by increasing the phagocytic activity of granulocytes, the activity of immunoglobulin-secreting cells in the blood, and the excretion of cytokines in lymphocytes [22]. The probiotic bacteria *Bifidobacterium* spp. and *L. acidophilus* reduce blood cholesterol by causing the deconjugation of bile salts, binding cholesterol to bacterial cell walls and contributing to the reduction of cholesterol biosynthesis [23,24]. Furthermore, *Bifidobacterium* and *Lactobacillus* have been proven to reduce obesity and insulin resistance by regulating metabolism. The prevention of gastrointestinal infections is a consequence of probiotics’ production of substances with antimicrobial properties, such as organic acids, mainly lactic and acetic acids, and through the stimulation of immune processes in the host. To induce a beneficial probiotic effect, the minimum therapeutic dose of a probiotic should be 10^6^–10^9^ CFU.

Due to growing consumer interest, products containing cultures of live probiotic microorganisms have become an essential commercial commodity, coming in various forms: tablets, sprays, liquids, suspensions, capsules, powder sachets, granules and in food [25]. Probiotics must be present in the food product in sufficient quantity before expiration, pass through the gastrointestinal tract, resist acidic and alkaline environments, and colonize the gut in sufficient numbers required to have a measurable beneficial effect [9,26]. Therefore, the quality of the final product is highly dependent on production processes, where procedures such as fermentation, the chemical composition of the growth environment, cell isolation, spray drying, freeze-drying and storage conditions such as temperature, humidity and pH are production factors that can affect microbial survival and growth [27,28,29]. Probiotic products are available as medicinal products, but most are categorized as foods or dietary supplements. In 2001, the Food and Agriculture Organization/World Health Organization [9] issued recommendations on the information that should be on the label of a probiotic food product: designation of genus, species and strain, minimum viable number of each probiotic strain at the end of shelf life, suggested serving size, which must provide an effective dose of probiotics related to the health claim, proper storage conditions and manufacturer’s contact information. The quality control process for probiotic food products should ensure that the probiotic content stated on the label corresponds to the actual content throughout the shelf life of the product while being free of contamination [25].

Optimal conditions for the growth of probiotic bacteria include a temperature of 30–40 °C and a pH of 5.5–6.2, but they can grow at a temperature of 2–53 °C [30] and a pH varying between 4.5 and 7.0, depending on the strain. However, culture conditions can affect growth kinetics parameters, such as the specific growth rate and the lag phase duration, which is the time bacteria adapt to a new medium and do not multiply [31]. The literature reports on the kinetics parameters of milk acidification by various strains of lactic acid bacteria, including probiotic strains, where the maximum acidification rate (Vmax) ranged from 15.9 to 18.9 × 10^−3^ upH/min for milk fermented by *L. bulgaricus*, *L. acidophilus*, *L. rhamnosus* and *B. animalis* ssp. *lactis* BB-12 with co-cultures of *S. thermophilus*. When 4% inulin was added to the milk, there was an increase in the time to reach Vmax, which ranged from 17.0 to 19.0 × 10^−3^ upH/min [32].

Probiotic bacteria cells are destroyed during freezing and aeration of the ice cream mixes and in storage; however, many researchers have shown that probiotic cultures have better viability in frozen products than in fermented milk [33,34,35]. The most commonly applied probiotic bacteria in food production, including ice cream, are *Lactobacillus* and *Bifidobacterium* [36,37]. The probiotic strains of these bacteria are potentially resistant to low temperatures during freezing, extremely low pH (1–4) in the stomach, the activity of digestive enzymes (bile salts and pancreatin) in the intestine, and toxic metabolites formed during digestion [38,39,40].

Prebiotics are substrates that pass through the esophagus and stomach, reaching the intestines in undigested form and providing an abundant energy source for intestinal microflora [26,41,42]. Research results indicate that the consumption of dietary fiber, particularly inulin and fructooligosaccharides (FOS), can stimulate growth and increase the activity of intestinal microflora, leading to the restoration of human health and intestinal colonization by probiotic bacteria [43]. It was also confirmed that these compounds improve mineral absorption and regulate lipid metabolism, and reduce the possibility of diseases, including cardiovascular disease, diabetes, constipation and colon cancer [44,45,46]. Moreover, these natural polysaccharides were proven to positively affect the ice cream-making process by improving gelation and controlling crystallization. Probiotics include apple fiber, which consists of pectin and insoluble cellulose [47]. Inulin can be safely used as a thickener and substitute for sugar and fat [48,49]. The use of various strains of probiotics and the use of inulin and apple fiber in ice cream manufacturing could affect the survival rate of probiotics in the human gastrointestinal tract. Therefore, it is essential to evaluate the survival of bacteria before and during passage through the gastrointestinal tract using an in vitro model digestion system [50].

Sheep milk ice cream combines the health-promoting properties of sheep milk with the beneficial effects of fiber and probiotics on human health. Studies on synbiotic sheep milk ice cream are important for ice cream manufacturers, consumers and nutritionists. These studies disprove of the myth of ice cream as a provider of empty calories consisting of sugar. Sheep milk ice cream provides a matrix rich in protein, fat and vitamins, further protecting probiotic cells. Therefore, it is crucial to show whether probiotic monocultures applied in the dairy industry can survive under simulated gastrointestinal conditions and induce a beneficial probiotic effect. The study aimed to evaluate the survival of five strains of probiotic bacteria, namely *Lacticaseibacillus casei* 431, *Lactobacillus acidophilus* LA-5, *Bifidobacterium animalis* ssp. *lactis* BB-12, *Lacticaseibacillus paracasei* L-26 and *Lacticaseibacillus rhamnosus*, by simulated in vitro digestion in ice cream manufactured from sheep milk, using different doses of apple fiber and inulin.

## 2. Materials and Methods

### 2.1. Materials

Enzymes and reagents: heat-stable α-amylase (TDF-100A, 24,975 U/mL; Sigma-Aldrich, St. Louis, MO, USA), mucin from the porcine stomach (type II; Sigma-Aldrich, St. Louis, MO, USA), pepsin from the porcine gastric mucosa (250 U/mg solid; Sigma-Aldrich, St. Louis, MO, USA), porcine bile extract (Sigma-Aldrich, St. Louis, MO, USA), pancreatin from the porcine pancreas (8×USP specifications; Sigma-Aldrich, St. Louis, MO, USA), di-sodium hydrogen phosphate anhydrous pure p.a. ≥99.0% (Na_2_HPO_4_; 141.96 g/mol; Chempur, Piekary Śląskie, Poland), di-potassium hydrogen phosphate (K_2_HPO_4_; 174.18 g/mol; Chempur, Piekary Śląskie, Poland), sodium chloride pure p.a. ≥99.9% (NaCl; 58.44 g/mol; Chempur, Piekary Śląskie, Poland), hydrochloric acid 12 mol (HCl, Chempur, Piekary Śląskie, Poland), sodium hydroxide 1 mol (NaOH, Chempur, Piekary Śląskie, Poland). All of the reagents used were of analytical reagent grade.

Materials used in the manufacture of ice cream mixtures: raw sheep milk was purchased from the farm “Owcza Zagroda” (Wyżne, Podkarpacie, Poland); inulin (Orafti HP, Oreye, Belgium); apple fiber (Aura Herbals Jarosław Paul, Sopot, Poland); and white sugar (Polish Sugar, Toruń, Poland). Five types of commercial freeze-dried starter cultures for direct vat set (*Lacticaseibacillus paracasei* L-26, *Lacticaseibacillus casei* 431, *Lactobacillus acidophilus* LA-5, *Lacticaseibacillus rhamnosus* and *Bifidobacterium animalis* ssp. *lactis* BB-12) were purchased from Chr. Hansen (Hoersholm, Denmark). These starter cultures are widely used by the dairy industry to produce probiotic fermented milk and are not genetically modified.

### 2.2. Ice Cream Manufacture

For each probiotic bacteria, 4 groups of mixtures with additives were prepared:

1. Sheep milk (89%) with sugar (11%);
2. Sheep milk (85%), sugar (11%), inulin (4%);
3. Sheep milk (85%), sugar (11%), inulin (1.5%), apple fiber (2.5%);
4. Sheep milk (85%), sugar (11%), apple fiber (4%).

Five groups of inoculum were prepared according to the method of Mituniewicz-Małek et al. [51] and Kowalczyk et al. [52]. Previously revived monocultures of probiotic bacteria, in the amount of 10 mg L^−1^ (*w/w*), were activated at 40 °C until the pH was 4.60 ± 0.10. Bacteria inoculums were approximately 9 log CFU bacteria g^−1^. The inoculum was added to ice cream mixes in the amount of 5% (*w/w*). Prior to adding inoculum, each mixture was homogenized with a homogenizer (CAT UNIDRIVE X 1000 D, Ballrechten-Dottingen, Germany) and then pasteurized at 85 °C, 1 min. After the heat treatment, the mixtures were cooled to 37 °C and fermented in an incubator (cooled incubator ILW 115, POL-EKO-Aparatura, Wodzisław Śląski, Poland) at 37 °C for ten hours, then cooled to 5 °C and conditioned at this temperature for 12 h. The ice cream mixtures were frozen in a DeLux 48,816 freezer (UNOLD AG, Hockeheim, Germany) for 40–50 min with freezing temperatures down to −22 °C. The ice cream was packed into 100 mL plastic cups and stored at −22 °C for seven days. The experiment was repeated three times. The ice cream was coded according to the probiotic bacteria type and the different fiber and inulin doses (Table 1).

### 2.3. In Vitro Digestion Process

In vitro gastrointestinal digestion was conducted according to the methods presented by Buniowska et al. [53] and Camelo-Silva et al. [54], with slight modifications. The process of simulated in vitro digestion of ice cream was carried out after seven days of storage at −22 °C. The in vitro digestion method simulates the digestion stages that occur in the mouth, stomach and small intestine.

The in vitro digestion model was initiated from the oral stage. 50 mL of each sample was transferred to a 100 mL dark glass bottle and then mixed with 5 mL of salivary enzyme solution. The salivary solution was obtained by mixing and dissolving 2.38 g of Na_2_HPO_4_, 0.19 g of K_2_HPO_4_, 8 g of NaCl, 100 mg/L of mucin and 150 mg/L α-amylase with enzymatic activity 200 U/L, solution per 1 L of distilled water. The mixture of dissolved ice cream and saliva was adjusted to pH 6.75 ± 0.20 with HCl (12 mol/L) or NaOH (1 mol/L) and incubated in a shaking water bath at 37 °C and 90 rpm for 10 min.

The stomach stage was initiated by adding 13.08 mg of pepsin to the sample after the oral stage, and the pH was reduced to 2.0 ± 0.20 by adding HCl (12 mol/L). The sample with digestive contents was placed in a shaking water bath for 2 h at 37 °C and 90 rpm.

To obtain the intestinal fraction, the oral and gastric contents were mixed with 5 mL pancreatin (4 g/L) and bile salt (25 g/L) at a pH value of 7.00 ± 0.20 (HCl 12 mol/L or NaOH 1 mol/L) and incubation was continued for another 2 h (37 °C, 90 rpm).

The survival rate (%) was determined in terms of viable colony counts of probiotic bacteria remaining in the intestinal content relative to the non-digested sample, according to Equation (1):

(1) $$\mathrm{Survival}\;\mathrm{rate}\;(\%)=\frac{Viable\;counts\;of\;probiotic\;bacteria\;\;in\;digested\;sample\times 100}{Viable\;counts\;of\;probiotic\;bacteria\;in\;non-digested\;sample}$$

### 2.4. Microbiological Analysis

The survival rates of probiotic strains: *L. paracasei* L-26, *L. casei* 431, *L. acidophilus* LA-5, *L. rhamnosus* and *Bifidobacterium* BB-12 were determined in ice cream before digestion and at each stage of simulated in vitro digestion (oral, stomach and intestinal stage) after seven days of storage at −22 °C. According to the method of Znamirowska et al. [55], 10 g of each sample was diluted in 90 mL of sterile peptone water solution (0.1%) (BTL Sp. z o.o., Łódź, Poland). Serial dilutions were prepared between 1 log cfu g^−1^ and 8 log cfu g^−1^. Inoculation was performed by the plate-deep method using MRS agar (Biocorp, Warsaw, Poland). A vacuum desiccator and GENbox anaer (Biomerieux, Warszawa, Poland) were used to maintain anaerobic conditions, while an Anaer indicator (Biomerieux, Warszawa, Poland) was used to monitor anaerobic conditions. The cultured probiotic colonies were counted using a colony counter (TYP J-3, Chemland, Stargard Szczeciński, Poland). The result was presented as log cfu g^−1^.

### 2.5. Statistical Analysis

From the obtained results, the mean and standard deviation were calculated using Statistica v. 13.1 (StatSoft, Tulsa, OK, USA). One-, two-, and three-way ANOVA were performed. The significance of differences between the mean values was verified with the Tukey test (*p* < 0.05).

The *p*-value for the ANOVA determining the effect of bacterial type and inulin and apple fiber on the survival of probiotic bacteria in the simulated digestive system is included in the Supplementary Material.

## 3. Results

The number of bacterial cells in the ice cream before digestion was determined to evaluate the effect of adding inulin and apple fiber on the viability of probiotic strains during gastrointestinal passage. The results indicating the viable counts of probiotic bacteria of *L. casei*, *L. acidophilus*, *L. paracasei*, *L. rhamnosus* and *Bifidobacterium* BB-12 are shown in Table 2.

Before digestion, the highest number of bacterial cells was determined in ice cream with *L. casei* (CLC, ILC, IFLC), while the lowest number was determined in ice cream with *L. acidophilus* (CLA, ILA). The number of probiotic bacteria cells in all tested ice cream groups before digestion exceeded 6 log CFU g^−1^, which qualifies this product as probiotic ice cream and initially guarantees a therapeutic effect. However, only the number of cells determined at the small intestine stage will accurately indicate probiotic activity.

During the freezing process, specific changes occur in ice cream manufacturing that cause the loss of metabolic characteristics due to changes in osmotic pressure in the cells. The ice crystals that form can mechanically damage the cell walls of probiotic bacteria. Adverse factors also include the effects of oxygen during the aeration of the mixture or during storage and high redox potential values [33]. However, substances that exhibit selective protective effects to reduce cell damage during freezing may be added to the ice mix. Cryoprotectants include milk, whey, prebiotics, saccharides and amino acids [56]. On the other hand, during product melting, chemical stressors, i.e., osmotic stress and high concentrations of components such as hydrogen ions, organic acids, oxygen and other components, can negatively affect probiotic cells. In our study, during the freezing and storage of ice cream and passage through the simulated digestive system, the addition of inulin, apple fiber, and sheep’s milk performed protective functions for the probiotics.

A three-way analysis of variance indicated that the number of bacterial cells before digestion was significantly affected by the type of bacteria, the interaction between the type of bacteria (*p* = 0.000), the interactions between bacterial type and inulin addition (*p* = 0.0003), and the interactions between bacterial type and apple fiber addition (*p* = 0.0001) (Table S1). It was also demonstrated that adding inulin to ice cream did not significantly increase the number of probiotic bacteria in ice cream compared to the control samples before digestion [57]. In ice cream fermented by *L. acidophilus*, it was shown that the addition of apple fiber significantly increased the number of viable cells before digestion, while in ice cream with *L. casei* 4%, the addition of apple fiber significantly reduced the number of cells of this probiotic.

The results in Table 2 show that at the end of the oral stage, there was no significant loss of probiotic viability in any ice cream group compared to the results obtained before digestion. These results are consistent with Melchior et al.’s [58] study, which also showed no significant effect of saliva activity on the viability of probiotic bacteria. Since the optimal pH for these probiotics is 6.5 to 7.5 and saliva is 6.5 to 7.0, the pH in this environment is suitable for bacterial cell survival [59]. Venema et al. [60] showed that the intake of probiotics before, during or after a meal can significantly affect their survival rate. Since human physiological conditions in the gastrointestinal tract vary depending on the time of probiotic ingestion, they interact differently with the intestinal lining, which dissolves depending on the pH of the stomach. The pH in the stomach before meal consumption (pH~2.0) is different from that during the meal (increasing to pH 5.5–7.0 within 2–3 h) and 1 h after meal (decreasing pH to 2.5–4.0) and depends on the age of the host. In this study, the pH was reduced to about 2.0 during the stomach stage. As is commonly known, strongly acidic conditions typical of the gastric environment can damage the cell membranes of DNA and proteins. Meanwhile, in the small intestine, the presence of bile salts can damage protein DNA through oxidative shock and low intracellular pH. These factors usually result in a substantial reduction in cell viability during digestion [58,61].

Among the control samples, the CBB12 samples with *Bifidobacterium* BB-12 and the CLC samples with *L. casei* 431 were found to be the most susceptible to the acid stress that occurs during gastric passage, with population reductions of 73.93% and 65.52% compared to the number of viable cells before digestion. Slightly smaller population reductions in the stomach (43.59%) were found in the CLR control sample with *L. rhamnosus*. Probiotics must survive in the stomach’s acidic environment if they reach the small intestine and colonize the host. *Lactobacillus* species are thought to be intrinsically acid resistant [62]. Although there are differences between species and strains, organisms generally show increased sensitivity at pH values below 3.0 [63,64]. Hence, acid tolerance is one of the desirable properties used to select potentially probiotic strains. The strong acidic environment in the stomach reduces the endogenous pH due to the intracellular accumulation of protons (H+ ions) and also affects the trans-membrane pH. Lactic acid and acetic acid could penetrate the cell membrane and dissociate to form H+, reducing cytoplasmic pH and promoting transport across the membrane. Acid stress can cause damage to the cell membrane, DNA and proteins. Thus, acid resistance is one of the selection criteria for probiotics [65,66]. The resistance to an acidic environmental reaction by lactobacilli is due to the presence of a constant gradient between extracellular and cytoplasmic pH. Cellular functions are inhibited, and cells die when the internal pH reaches a critical limit [67]. The enzyme F0F1-ATPase is used by gram-positive organisms to protect themselves from acidic environmental conditions. F0F1-ATPase, induced at low pH, can increase intracellular pH at low extracellular pH [68]. It was concluded that the best survival rate of the gastric stage among the control samples was CBB12 with *Bifidobacterium* BB-12, where a population reduction of only 26.0% was found compared to the count before digestion. Furthermore, it was found that the addition of inulin to IBB12 ice cream increased gastric juice tolerance and reduced strain reduction to 16.7% compared to the number of bacterial cells before digestion. For IFBB12 samples with *Bifidobacterium* BB-12, replacing part of the inulin with apple fiber resulted in increased survival in the gastric stage and a poor reduction in the bacterial population of only 15.6% compared to the samples before digestion. However, the complete replacement of inulin with apple fiber in the IFBB12 ice cream resulted in a population reduction of 22.4%, but the survival rate was still 3.6% higher than in the CBB12 control sample. The obtained results indicate that the addition of these fibers to ice cream with *Bifidobacterium* BB-12 increases the survival of this strain in model gastric juice.

In the gastric stage, the positive effect of the addition of inulin and apple fiber was also demonstrated for ice cream samples fermented with *L. casei*, *L. acidophilus* and *L. paracasei*. The best survival of probiotics in the stomach was observed for ice cream samples with inulin partially replaced by IFLC apple fiber (population reduction of 24.6%, 29.6% and 48.6%, respectively, compared to live cells before digestion). This study demonstrated the highest sensitivity to simulated gastric stress for ice cream samples with *L. rhamnosus*. At the gastric stage, there was a reduction of 56.4% of the population in the CLR ice cream compared to the cell counts before digestion. Adding fibers to the samples with this strain resulted in an even higher 1–3% population reduction at the stomach stage compared to the CLR control sample. In the FLC, FLA, FBB12 and FLP ice cream samples, the 4% addition of apple fiber (without inulin) was found to increase bacterial survival more effectively than the presence of a combination of inulin (2.5%) and apple fiber (1.5%) in the gastric juice.

Numerous studies confirm the improved survival rate of microencapsulated probiotics under simulated in vitro digestion [69,70] and report that, in a low pH environment, positively charged milk protein molecules repel each other, resulting in the dissolution of microcapsules and loss of bacterial protection. In addition, Afzaal et al.’s [71] study confirmed that a low pH (2.0) contributed to lower numbers of *L. casei* in simulated gastric juice. A faster reduction in cell counts (from 10.79 to 5.48 log10 cfu/mL) was observed for ice cream samples with free probiotics compared to ice cream, where probiotics were encapsulated (10.72 to 7.65 log10 cfu/mL). Other studies indicate that the addition of inulin to microencapsulated probiotics provides protection upon passage through the gastrointestinal tract [72].

The intestinal juice is secreted at a rate of 0.7 L per day. Its pH is around 8 and the concentration of mineral salts is about 0.5%. The factors determining the survival of bacteria in this section of the digestive tract are bile and the presence of enzymes. The second barrier to the survival of lactic fermentation bacteria in the gastrointestinal tract is bile acids produced in the liver from cholesterol and secreted into the duodenum. The concentration of bile acid salts fluctuates between 0.15% and 0.5%. Bile secreted into the digestive tract plays an important role in emulsifying lipids, especially since the fat content in the ice cream evaluated ranged from 6.2% to 6.4%. Furthermore, bile has the ability to affect phospholipids and cell membrane proteins and thus disrupt the cellular homeostasis of microorganisms [73,74]. Bile salts exhibit potent antimicrobial activity by changing the conformation of cell membrane proteins and lipids, resulting in altered membrane integrity and permeability. In addition, bile salts induce the production of free radicals, which cause DNA damage [15]. However, many strains of probiotic bacteria have well-developed bile resistance mechanisms [75]. In addition, food components can protect probiotic strains from the negative effects of digestive juices. Microbial adhesion can also be affected by the culture method, more specifically, the composition of the culture medium, the number of bacteria and the incubation time [76]. Lebeer et al. [76] showed that limiting glucose availability in the medium affected biofilm formation by *L. rhamnosus* GG.

In our study, the highest sensitivity to simulated intestinal stress was found for *Bifidobacterium* BB-12 due to a reduction in survival from 12.7–31.0% compared to the high number of cells determined in the stomach. It should be mentioned that Ruas-Madiedo et al. [77] detected EPS production by *Bifidobacterium* in the presence of bile and explained that EPS probably poses a defense mechanism against this toxic compound. Ruiz et al. [78] investigated the effect of bile on the fatty acid composition and membrane characteristics of *B. animalis* IPLA 4549 and its mutant with acquired bile resistance, *B. animalis* 4549dOx. According to these authors’ studies, bile adaptation caused *B. animalis* 4549dOx to decrease membrane fluidity and protein: phospholipid ratio, as well as alter the fatty acid composition of the cell. Unfortunately, our study does not confirm the high tolerance of the *Bifidobacterium* strain BB-12 to bile.

Lower survival in the small intestine was also found for *L. casei*, which was lower by 6.1–21.9% compared to the number of bacteria determined in the stomach (Table 2). Our study confirmed that the 4% addition of inulin to ice cream increased the survival rates of *L. casei* and *Bifidobacterium* BB-12 under simulated intestinal juice with bile. The highest survival rate in the small intestine stage was reported in ice cream with *L. paracasei* and *L. rhamnosus*, as an increase in viable cells was observed in the small intestine compared to their count in the stomach. It should also be mentioned that good tolerance was found in the small intestine stage for the control sample with CLA and FLA apple fiber fermented by *L. acidophilus* compared to the number of cells of the strain determined in the stomach.

According to other authors’ studies, the survival rate of probiotic strains gradually decreased during in vitro passage through the stomach and small intestine [79,80]. In our study, the survival rate and tolerance of the transit of strains were evaluated using simulated in vitro digestion. When comparing the number of viable cells before digestion to the number of viable cells in the gut, we found the highest strain survival rate in the CLA control ice cream with *L. acidophilus* (82.7%). Presumably, the whey proteins in sheep milk contributed to better tolerance of *L. acidophilus* to the simulated digestive conditions of the gastrointestinal tract. In the study by de Figueiredo Furtado et al. [81], the physicochemical conditions of the gastric environment caused aggregation of fat droplets and partial hydrolysis of proteins, considering that whey proteins were resistant to gastric conditions. However, all proteins were extensively hydrolyzed after intestinal digestion, absorbing intestinal juices.

In ice cream with *L. acidophilus*, the addition of inulin and apple fiber resulted in a 11.1–31.4% reduction in the survival of this strain compared to the CLA sample. Meanwhile, the lowest survival rate was found in the CLR control ice cream with *L. rhamnosus*. As shown by the results in Table 2, the addition of inulin increased the survival rate of *L. rhamnosus* by 10.8% compared to the CLR control sample under simulated in vitro digestion conditions. An even higher survival rate (about 22%) after inulin addition compared to the control sample was found for the *Bifidobacterium* BB-12 strain. This is consistent with the findings of Tarifa et al. [47], where the survival rate of free and encapsulated cells of *L. casei* and *L. rhamnosus* in the absence of inulin significantly decreased, between 60% and 90%, after passage through the gastrointestinal tract, showing a 62.2% reduction in the *Lactobacillus* population in simulated in vitro digestion. Furthermore, Hu et al. [59] showed a 62.2% reduction in the *Lactobacillus* population in simulated in vitro digestion. In contrast, the addition of only apple fiber improved the survival of *Bifidobacterium* BB-12 under simulated in vitro digestion by only about 6% compared to the control. Similarly, the survival rate of *L. casei, L. paracasei,* and *L. acidophilus* was also highly affected by the 4% addition of apple fiber, where its increase under gastrointestinal passage conditions was determined to range from 5.7–11.2% compared to their control counterparts. In apple fiber, soluble pectin represents 40% of the content, and about half is insoluble cellulose [82]. Pectin and cellulose show less sensitivity to chemical agents and higher resistance to the gastric environment than alginate. Pectin and cellulose have been found to be suitable mucoadhesive materials [83], which can prolong the residence time and exposure time. Furthermore, they exist as aggregates of macromolecules in an acidic environment and are resistant to proteases and amylases, which are active in the upper gastrointestinal tract, which may explain the better survival of some bacterial strains [84].

Krasaekoopt and Bhandari [85] also found that when the synbiotic matrix absorbed bile salt in the intestine, an insoluble complex between the positive calcium ion and the negative cholate was formed electrostatically. It reduces the diffusion of bile salt into the matrix and inhibits the interaction between bile salt and trapped cells. Moreover, Li and Zhang [86] reported for *L. rhamnosus* that pectin cross-links with Ca^2+^ to protect probiotic cells from acid inactivation. *Bifidobacterium* ssp. show poor pectin degradation but are adapted to degrade side chains from arabinate and galactan [87]. In a subsequent study, pectin encapsulation was found to improve the survivability of *L. rhamnosus* in gastric environments at a very low pH [88]. Gebara and Chaves [89] described a similar study in which pectin-encapsulated *Lactobacillus acidophilus* showed less reduction (1.51 log cycles) than unencapsulated cells (3.54 log cycles) when incubated in gastric and intestinal juices. Additionally, a study by Tarifa et al. [47] showed that *L. casei* and *L. rhamnosus* coated with pectin and inulin could improve viability under gastrointestinal conditions compared to free cells. At the end of the sequence, the survival rate of *L. casei* 393 was 51.00%, and the survival rate for *L. rhamnosus* was 61.00%.

In our study, in the small intestine stage, only five ice cream groups (ILA, CBB12, IFBB12, CLR and FLR) had bacterial cell counts below 6 log cfu g^−1^, but higher than 5 log cfu g^−1^. In the remaining ice cream groups, the number of viable bacterial cells ranged from 6.11 to 8.88 log cfu g^−1^, which qualifies them as probiotic ice cream and guarantees a therapeutic effect.

The three-way analysis of variance indicated that the survival rate and tolerance to conditions in the small intestine were significantly influenced by all the factors analyzed (inulin, apple fiber, type of bacteria) and their interactions.

The gastrointestinal tract passage might not be sufficient for good intestinal colonization [90]. Since the adhesion of bacteria to the intestinal epithelium affects their residence time in the gastrointestinal tract, this ability is considered an important criterion when selecting probiotic strains [91]. Bacterial binding to intestinal epithelial cells is not regulated by one specific molecule but by several different factors. These include cell wall elements, various proteins, the presence of intestinal mucus and environmental conditions.

Adhesion is a process that allows microorganisms to adhere to other cells or surfaces [15]. Structures located on the surface of microbial cells directly affect this process. Factors affecting the adhesion process of the probiotic bacterium *Lactobacillus* ssp. can be divided into protein, non-protein, environmental, aggregation capacity and hydrophobicity. Factors such as bile salts, low pH, digestive enzymes or oxidative and osmotic stress affect the cell wall properties of lactobacilli and, thus, their adhesion abilities. Tuomola et al. [92] showed that applying digestive enzymes (trypsin and pepsin) reduced *L. acidophilus* LA-1 adhesion, indicating that the outer protein layer of the cell wall plays an essential role in cell adhesion. Moreover, Lim and Ahn [93], who studied the effect of proteolytic enzymes on the adhesion capacity of seven *Lactobacillus* strains, showed that *L. plantarum* GK81, *L. acidophilus* GK20, and *L. paracasei* GK74 strains, after incubation with pepsin, protease and trypsin, showed significantly lower adhesion to Caco-2 cells (immortalized cell line of human colorectal adenocarcinoma cells). It was also observed that the adhesion of probiotic strains is related to the growth phase of the microorganisms and that cells in the logarithmic growth phase adhere better than cells in the stationary phase [76].

Additionally, for probiotic bacteria to benefit the host, they need to reach a sufficient mass. Therefore, the ability to aggregate is desirable among probiotic strains [94]. The formation of aggregates between cells of the same strain is autoaggregation or self-aggregation, while coaggregation occurs between different strains and even species [95]. These properties are essential in colonizing various environments, especially the intestines, oral cavity and genitourinary tract, by probiotic bacteria. Autoaggregation of probiotic strains is essential in the process of microbial adhesion to the intestinal epithelium [15].

## 4. Conclusions

The popularity of frozen desserts continues to grow with new varieties and flavors. Food manufacturers can provide consumers with healthier probiotic- and prebiotic-providing frozen dessert options. An additional nutritional benefit would be for food manufacturers to provide a product that qualifies as probiotic ice cream and pre-guarantees a therapeutic effect, similar to the probiotic strains provided with drugs and food supplements. Sheep milk functional ice cream is a food product that contains valuable bioactive ingredients that offer health benefits beyond their nutritional value. The bioactive ingredients are synbiotics, a combination of probiotics and prebiotics, which are generally important for gut health.

The conducted studies clearly indicate that sheep milk ice cream could provide a suitable matrix for the delivery of probiotics from commercial dairy cultures and prebiotics and could contribute to intestinal homeostasis. The results indicated that in the small intestinal phase, all groups had a higher probiotic content than 5 log cfu g^−1^, demonstrating a good survival rate.

Evaluating the effect of fiber addition on the survival of probiotic bacterial strains in the gastrointestinal tract would be helpful in developing new and innovative products with enhanced health properties tailored to the needs of the intestinal ecosystem.

## Figures and Tables

**Table 1:** Ice cream experimental groups.

Bacterial Strain	Control Group	Group with 4% Inulin	Group with 2.5% Inulin and 1.5% Apple Fiber	Group with 4% Fiber
*Lacticaseibacilluscasei* 431	CLC	ILC	IFLC	FLC
*Lactobacillus acidophilus* LA-5	CLA	ILA	IFLA	FLA
*Lacticaseibacillus paracasei* L-26	CLP	ILP	IFLP	FLP
*Lacticaseibacillus* *rhamnosus*	CLR	ILR	IFLR	FLR
*Bifidobacterium animalis* ssp. *lactis* BB-12	CBB12	IBB12	IFBB12	FBB12

**Table 2:** Survival [%] and viable counts of probiotic bacteria in ice cream (log cfu g^−1^) before and during simulated in vitro digestion.

Group of Ice Cream	Viable Counts of Probiotic Bacteria in Ice Cream, Log cfu g^−1^				Survival Rate, %
	Before Digestion	Simulated In Vitro Digestion Stage			
		Oral	Stomach	Small Intestine	
CLC	12.18 ^cB^ ± 0.40	12.12 ^cB^ ± 0.39	7.98 ^bA^ ± 0.57	6.94 ^aA^ ± 0.12	56.97
ILC	12.14 ^cB^ ± 0.32	12.03 ^cB^ ± 0.34	8.84 ^bB^ ± 0.27	7.81 ^aC^ ± 0.28	64.33
IFLC	12.57 ^cB^ ± 0.37	12.13 ^cB^ ± 0.26	9.47 ^bC^ ± 0.32	7.39 ^aB^ ± 0.18	58.79
FLC	11.80 ^cA^ ± 0.33	11.48 ^cA^ ± 0.13	8.15 ^bAB^ ± 0.52	7.65 ^aBC^ ± 0.16	64.83
CLA	10.73 ^cA^ ± 0.73	10.05 ^cA^ ± 0.10	5.01 ^aA^± 0.15	8.88 ^bD^ ± 0.21	82.75
ILA	10.24 ^bA^ ± 0.46	10.20 ^bA^ ± 0.44	5.26 ^aA^ ± 0.58	5.16 ^aA^ ± 0.23	50.39
IFLA	11.46 ^cB^ ± 0.61	11.22 ^cB^ ± 0.46	8.06 ^bC^ ± 0.24	7.02 ^aB^ ± 0.79	61.25
FLA	11.85 ^cB^ ± 0.96	11.78 ^cB^ ± 0.61	6.84 ^aB^ ± 0.29	8.49 ^bC^ ± 0.17	71.64
CBB12	11.47 ^cA^ ± 0.57	11.34 ^cA^ ± 0.51	8.48 ^bA^ ± 0.21	5.83 ^aA^ ± 0.14	50.82
IBB12	11.13 ^cA^ ± 0.56	10.83 ^cA^ ± 0.47	9.27 ^bB^ ± 0.22	8.09 ^aC^ ± 0.10	72.68
IFBB12	11.00 ^cA^ ± 0.35	10.61 ^cA^ ± 0.26	9.28 ^bB^ ± 0.18	5.91 ^aA^ ± 0.09	53.72
FBB12	11.17 ^cA^ ± 0.45	11.13 ^cA^ ± 0.69	8.66 ^bA^ ± 0.24	6.32 ^aB^ ± 0.16	56.58
CLP	11.28 ^cA^ ± 0.59	11.31 ^cA^ ± 0.58	5.04 ^aA^ ± 0.44	6.31 ^bA^ ± 0.39	55.93
ILP	11.37 ^bA^ ± 0.89	11.39 ^bA^ ± 0.50	5.74 ^aB^ ± 0.27	6.16 ^aA^ ± 0.31	54.17
IFLP	11.28 ^bA^ ± 0.60	11.19 ^bA^ ± 0.48	5.79 ^aB^ ± 0.22	6.11 ^aA^ ± 0.23	54.16
FLP	11.37 ^cA^ ± 0.88	11.35 ^cA^ ± 0.75	6.47 ^aC^ ± 0.27	7.64 ^bB^ ± 0.15	67.19
CLR	10.99 ^cA^ ± 0.42	10.98 ^cA^ ± 0.33	4.79 ^aA^ ± 0.24	5.21 ^bA^ ± 0.10	47.40
ILR	11.21 ^cA^ ± 0.36	11.12 ^cA^ ± 0.63	4.74 ^aA^ ± 0.21	6.53 ^bC^ ± 0.19	58.25
IFLR	11.21 ^cA^ ± 0.38	11.11 ^cA^ ± 0.45	4.54 ^aA^ ± 0.30	6.50 ^bC^ ± 0.39	57.98
FLR	11.25 ^cA^ ± 0.53	11.20 ^cA^ ± 0.29	4.77 ^aA^ ± 0.14	5.73 ^bB^ ± 0.26	50.93

Mean ± standard deviation; *n* (for each trial) = 9; a–c—mean values denoted in rows by different letters differ statistically significantly at *p* ≤ 0.05; A–D—mean values in columns obtained for a single bacterial strain denoted by different letters differ significantly *p* ≤ 0.05; CLC: control sample with *L. casei* 431; ILC: sample with 4% inulin and *L. casei* 431; IFLC: sample with 2.5% inulin and 1.5% apple fiber with *L. casei* 431; FLC: sample with 4% fiber and *L. casei* 431; CLA: control sample with *L. acidophilus;* ILA: sample with 4% inulin and *L. acidophilus*; IFLA: sample with 2.5% inulin and 1.5% apple fiber with *L. acidophilus*; FLA: sample with 4% fiber and *L. acidophilus*; CBB12: control sample with *Bifidobacterium* BB12; IBB12: sample with 4% inulin and *Bifidobacterium* BB12; IFBB12: sample with 2.5% inulin and 1.5% apple fiber with *Bifidobacterium* BB12; FBB12: sample with 4% fiber and *Bifidobacterium* BB12; CLP: control sample with *L. paracasei* L-26; ILP: sample with 4% inulin and *L. paracasei* L-26; IFLP: sample with 2.5% inulin and 1.5% apple fiber with *L. paracasei* L-26; FLP sample with 4% fiber and *L. paracasei* L-26; CLR: control sample with *L. rhamnosus*; ILR: sample with 4% inulin and *L. rhamnosus*; IFLR: sample with 2.5% inulin and 1.5% apple fiber with *L. rhamnosus*; FLR: sample with 4% fiber and *L. rhamnosus.*

## Data Availability

The original data presented in the study are included in the article, further inquiries can be directed to the corresponding author.

## Supplementary Materials

The following supporting information can be downloaded at: https://www.mdpi.com/article/10.3390/nu14214454/s1.

## References

[1] Ares G., Giménez A., Gámbaro A. (2009). Consumer-perceived healthiness and willingness to try functional milk desserts. Influence of ingredient, ingredient name and health claim. Food Qual. Prefer..

[2] Ranadheera C.S., Naumovski N., Ajlouni S. (2018). Non-bovine milk products as emerging probiotic carriers: Recent developments and innovations. Curr. Opin. Food Sci..

[3] Rasika D.M.D., Munasinghe M.A.D.D., Vidanarachchi J.K., da Cruz A.G., Ajlouni S., Ranadheera C.S. (2020). Probiotics and prebiotics in non-bovine milk. Adv. Food Nutr. Res..

[4] Chia J., Burrow K., Carne A., McConnell M., Samuelsson L., Day L., Young W., Bekhit A.E.-D., Watson R.R., Collier R.J., Preedy V. (2017). Minerals in Sheep milk. Nutrients in Milk and Their Implications on Health and Disease.

[5] Dominguez D.C. (2004). Calcium signalling in bacteria. Mol. Microbiol..

[6] Purwandari U., Vasiljevic T. (2012). Microbial growth, EPS concentration and textural properties of fermented milk supplemented with calcium and whey protein analysed using response surface methodology. Int. Food Res. J..

[7] Ng I.S., Xue C. (2017). Enhanced exopolysaccharide production and biological activity of *Lactobacillus rhamnosus* ZY with calcium and hydrogen peroxide. Process Biochem..

[8] Pu M., Storms E., Chodur D.M., Rowe-Magnus D.A. (2020). Calcium-dependent site-switching regulates expression of the atypical *iam* pilus locus in *Vibrio vulnificus*. Environ. Microbiol..

[9] FAO, WHO (2001). Joint Expert Consultation Report: Evaluations of Health and Nutritional Properties of Probiotics in Food Including Powder Milk and Live Lactic Acid Bacteria, Cordoba, Argentina. http://www.fao.org/documents/pub_dett.asp?lang=en&pub_id=61756/.

[10] Hill C., Guarner F., Reid G., Gibson G.R., Merenstein D.J., Pot B., Morelli L., Berni Canani R., Flint H.J., Salminen S. (2014). The International Scientific Association for Probiotics and Prebiotics consensus statement on the scope and appropriate use of the term probiotic. Nat. Rev. Gastroenterol. Hepatol..

[11] Dale H.F., Rasmussen S.H., Asiller Ö.Ö., Lied G.A. (2019). Probiotics in irritable bowel syndrome: An up-to-date systematic review. Nutrients.

[12] Barichella M., Pacchetti C., Bolliri C., Cassani E., Lorio L., Pusani C., Pinelli G., Privitera G., Cesari I., Faierman S.A. (2016). Probiotics and prebiotic fiber for constipation associated with Parkinson disease: An RCT. Neurology.

[13] Toejing P., Khampithum N., Sirilun S., Chaiyasut C., Lailerd N. (2021). Influence of *Lactobacillus paracasei* HII01 Supplementation on Glycemia and Inflammatory biomarkers in Type 2 Diabetes: A randomized clinical trial. Foods.

[14] Lv T., Ye M., Luo F., Hu B., Wang A., Chen J., Yan J., He Z., Chen F., Qian C. (2021). Probiotics treatment improves cognitive impairment in patients and animals: A systematic review and meta-analysis. Neurosci. Biobehav. Rev..

[15] Paliwoda A., Nowak A. (2017). Czynniki warunkujące zdolności adhezyjne bakterii z rodzaju *Lactobacillus* (In Polish). Factors determing the adhesive capacity of Lactobacillus bacteria. Post. Mikrobiol.-Adv. Microbiol..

[16] Umu Ö.C.O., Rudi K., Diep D.B. (2017). Modulation of the gut microbiota by prebiotic fibres and bacteriocins. Microb. Ecol. Health Dis..

[17] Gajewska J., Błaszczyk M.K. (2012). Probiotyczne Bakterie fermentacji mlekowej (LAB) (In Polish). Probiotic Lactic Fermentation Bacteria (LAB). Post. Mikrobiol..

[18] Castro-Bravo N., Wells J.M., Margolles A., Ruas-Madiedo P. (2018). Interactions of surface exopolysaccharides from *Bifidobacterium* and *Lactobacillus* within the intestinal environment. Front. Microbiol..

[19] Kumar M., Verma V., Nagpal R., Kumar A., Gautam S.K., Behare P.V., Grover C.R., Aggarwal P.K. (2011). Effect of probiotic fermented milk and chlorophyllin on gene expressions and genotoxicity during AFB1-induced hepatocellular carcinoma. Gene.

[20] Haskard C., Binnion C., Ahokas J. (2000). Czynniki wpływające na sekwestrację aflatoksyny przez *Lactobacillus rhamnosus* GG (In Polish). Factors affecting aflatoxin sequestration by *Lactobacillus rhamnosus* GG. Chem. Biol. Interact..

[21] Femia A.P., Luceri C., Dolara P., Giannini A., Biggeri A., Salvadori M., Clune Y., Collins K.J., Paglierani M., Caderni G. (2002). Antitumorigenic activity of the prebiotic inulin enriched with oligofructose in combination with the probiotics *Lactobacillus rhamnosus* and *Bifidobacterium lactis* on azoxymethane-induced colon carcinogenesis in rats. Carcinogenesis.

[22] Nagpal R., Kumar A., Kumar M., Behare P.V., Jain S., Yadav H. (2012). Probiotics, their health benefits and applications for developing healthier foods: A review. FEMS Microbiol. Lett..

[23] Pereira D.I.A., Gibson G.R. (2002). Cholesterol assimilation by lactic acid bacteria and *bifidobacteria* isolated from the human gut. Appl. Environ. Microbiol..

[24] Ishimwe N., Daliri E.B., Lee B.H., Fang F., Du G. (2015). The perspective on cholesterol-lowering mechanisms of probiotics. Mol. Nutr. Food Res..

[25] Kolaček S., Hojsak I., Berni-Canani R., Guarino A., Indrio F., Orel R., Pot B., Shamir R., Szajewska H., Vandenplas Y. (2017). Commercial Probiotic Products: A Call for Improved Quality Control. A Position Paper by the ESPGHAN Working Group for Probiotics and Prebiotics. J. Pediatr. Gastroenterol. Nutr..

[26] Gibson G.R., Hutkins R., Sanders M.E., Prescott S.L., Reimer R.A., Salminen S.J., Scott K., Stanton C., Swanson K.S., Cani P.D. (2017). Expert consensus document: The international scientific association for probiotics and prebiotics (ISAPP) consensus statement on the definition and scope of prebiotics. Nat. Rev. Gastroenterol. Hepatol..

[27] Auclair J., Frappier M., Millette M. (2015). Lactobacillus acidophilus CL1285, Lactobacillus casei LBC80R, and Lactobacillus rhamnosus CLR2 (Bio-K+): Characterization, Manufacture, Mechanisms of Action, and Quality Control of a Specific Probiotic Combination for Primary Prevention of Clostridium difficile Infection. Clin. Infect. Dis..

[28] Grześkowiak Ł., Isolauri E., Salminen S., Gueimonde M. (2011). Manufacturing process influences properties of probiotic bacteria. Br. J. Nutr..

[29] Nivoliez A., Camares O., Paquet-Gachinat M., Bornes S., Forestier C., Veisseire P. (2012). Influence of manufacturing processes on in vitro properties of the probiotic strain *Lactobacillus rhamnosus* Lcr35^®^. J. Biotechnol..

[30] König H., Berkelmann-Löhnertz B., König H., Gottfried U., Fröhlich J. (2017). Maintenance of wine-associated microorganisms. Biology of Microorganisms on Grapes, in Must and in Wine.

[31] Śliżewska K., Chlebicz-Wójcik A. (2020). Growth Kinetics of Probiotic *Lactobacillus* Strains in the Alternative, Cost-Efficient Semi-Solid Fermentation Medium. Biology.

[32] Oliveira R.P.S., Perego P., Converti A., Oliveira M.N. (2009). Growth and acidification performance of probiotics in pure culture and co-culture with *Streptococcus thermophilus*: The effect of inulin. LWT Food Sci. Technol..

[33] Mohammadi R., Mortazavian A.M., Khosrokhavar R., Cruz da Gomes A. (2011). Probiotic ice cream: Viability of probiotic bacteria and sensory properties. Ann. Microbiol..

[34] Hekmat S., McMahon D. (1992). Survival of *Lactobacillus acidophilus* and *Bifidobacterium bifidum* in ice cream for use as a probiotic food. J. Dairy Sci..

[35] Kailasapathy K., Sultana K. (2003). Survival of β-D-galactosidase activity of encapsulated and free *Lactobacillus acidophilus* and *Bifidobacterium lactis* in ice cream. Aust. J. Dairy Technol..

[36] Muninathan C., Guruchandran S., Viswanath Kalyan A.J., Ganesan N.D. (2021). Microbial exopolysaccharides: Role in functional food engineering and gut-health management. Int. J. Food Sci. Technol..

[37] Lee Y.K., Salminen S. (2009). Handbook of Probiotics and Prebiotics.

[38] Sangami R., Sri S.R. (2017). Emerging trends in improving viability, advanced stability techniques and health claims of healthy microbiome—The probiotics. Int. J. Curr. Microbiol. Appl. Sci..

[39] Ranadhera S., Evans C., Adams M.C., Baines K.S. (2012). In vitro analysis of gastrointestinal tolerance and intestinal cell adhesion of probiotics in goat’s milk ice cream and yogurt. Food Res. Int..

[40] Carvalho Lima K.G., Kruger M.F., Behrens J., Destro M.T., Landgraf M., Franco B.D.G.M. (2009). Evaluation of culture media for enumeration of *Lactobacillus acidophilus*, *Lactobacillus casei* and *Bifidobacterium animalis* in the presence of *Lactobacillus delbrueckii* subsp. *bulgaricus* and *Streptococcus thermophilus*. LWT–Food Sci. Technol..

[41] Qi X., Al-Ghazzewi F.H., Tester R.F. (2018). Dietary fiber, gastric emptying, and carbohydrate digestion: A mini-review. Starch-Stärke.

[42] Zhang H., Li Z., Zhang L., Lai P.F.H., Tian Y., Cui S.W., Ai L. (2021). Effects of soluble dietary fibers on the viscosity property and digestion kinetics of corn starch digesta. Food Chem..

[43] Pabari K., Pithva S., Kothari C., Purama R.K., Kondepudi K.K., Vyas B.R.M., Kothari R., Ambalam P. (2020). Evaluation of Probiotic Properties and Prebiotic Utilization Potential of *Weissella paramesenteroides* Isolated From Fruits. Probiotics Antimicrob. Proteins.

[44] Cai Y., Folkerts J., Folkerts G., Maurer M., Braber S. (2020). Microbiota-dependent and -independent effects of dietary fibre on human health. Br. J. Pharmacol..

[45] Ahmad A.M.R., Ahmed W., Iqbal S., Javed M., Rashid S., Haq U.L. (2021). Prebiotics and iron bioavailability? Unveiling the hidden association—A review. Trends Food. Sci. Technol..

[46] Rossi M., Corradini C., Amaretti A., Nicolini M., Pompei A., Zano N.S., Matteuzzi D. (2005). Fermentation of Fructooligosaccharides and Inulin by *Bifidobacteria*: A Comparative Study of Pure and Fecal Cultures. Appl. Environ. Microb..

[47] Tarifa M.C., Piqueras C.M., Genovese D.B., Brugnoni L.I. (2021). Microencapsulation of *Lactobacillus casei* and *Lactobacillus rhamnosus* in pectin and pectin-inulin microgel particles: Effect on bacterial survival under storage conditions. Int. J. Biol. Macromol..

[48] Genovese A., Balivo A., Salvati A., Sacchi R. (2022). Functional ice cream health benefits and sensory implications. Food Res. Int..

[49] Man S., Liu T., Yao Y., Lu Y., Ma L., Lu F. (2021). Friend or foe? The roles of inulin-type fructans. Carbohydr. Polym..

[50] Jacobsen N.M.Y., Caglayan I., Caglayan A., Bar-Shalom D., Mullertz A. (2020). Achieving delayed release of freeze-dried probiotic strains by extrusion, spheronization and fluid bed coating—Evaluated using a three-step in vitro model. Int. J. Pharm..

[51] Mituniewicz-Małek A., Ziarno M., Dmytrów I., Balejko J. (2017). Short Communication: Effect of the Addition of *Bifidobacterium* Monocultures on the Physical, Chemical, and Sensory Characteristics of Fermented Goat Milk. J. Dairy Sci..

[52] Kowalczyk M., Znamirowska A., Pawlos M., Buniowska M. (2022). The Use of Olkuska Sheep Milk for the Production of Symbiotic Dairy Ice Cream. Animals.

[53] Buniowska M., Carbonell-Capella J.M., Frigola A., Esteve M.J. (2017). Bioaccessibility of bioactive compounds after non-thermal processing of an exotic fruit juice blend sweetened with Stevia rebaudiana. Food Chem..

[54] Silva C.C., da Silva Barros E.L., Verruck S., Maran B.M., Canella M.H.M., Esmerino E.A., Ramon Silva R., Prudencio E.S. (2022). How ice cream manufactured with concentrated milk serves as a protective probiotic carrier? An in vitro gastrointestinal assay. Food Sci. Technol..

[55] Znamirowska A., Szajnar K., Pawlos M. (2021). Effect of Vitamin C Source on Its Stability during Storage and the Properties of Milk Fermented by *Lactobacillus rhamnosus*. Molecules.

[56] Szosland-Fałtyn A.M. (2007). Lody probiotyczne–zdrowe łakocie. (In Polish). Probiotic ice cream–healthy treats. Przemysł Spożywczy.

[57] Ahmad I., Khalique A., Shahid M.Q., Ahid Rashid A., Faiz F., Ikram M.A., Ahmed S., Imran M., Khan M.A., Nadeem M. (2020). Studying the Influence of Apple Peel Polyphenol Extract Fortification on the Characteristics of Probiotic Yoghurt. Plants.

[58] Melchior S., Marino M., Innocente N., Calligaris S., Nicoli M.C. (2020). Effect of different biopolymer-based structured systems on the survival of probiotic strains during storage and in vitro digestion. J Sci. Food Agric..

[59] Hu X., Liu C., Zhang H., Hossen Md A., Sameen D.E., Dai J., Qin W., Liu Y., Li S. (2021). In vitro digestion of sodium alginate/pectin co-encapsulated *Lactobacillus bulgaricus* and its application in yogurt bilayer beads. Int. J. Biol. Macromol..

[60] Venema K., Verhoeven J., Verbruggen S., Espinosa L., Courau S. (2019). Probiotic survival during a multi-layered tablet development as tested in a dynamic, computer-controlled in vitro model of the stomach and small intestine (TIM-1). Lett. Appl. Microbiol..

[61] Amund O.D. (2016). Exploring the relationship between exposure to technological and gastrointestinal stress and probiotic functional properties of lactobacilli and bifidobacteria. Can. J. Microbiol..

[62] Tannock G.W. (2004). A special fondness for lactobacilli. Appl. Environ. Microbiol..

[63] Jin L.Z., Ho Y.W., Abdullah N., Jalaludin S. (1998). Acid and bile tolerance of *Lactobacillus* isolated from chicken intestine. Lett. Appl. Microbiol..

[64] Wu C.H., Hsueh Y.H., Kuo J.M., Liu S.J. (2018). Characterization of a Potential Probiotic *Lactobacillus brevis* RK03 and Efficient Production of γ-Aminobutyric Acid in Batch Fermentation. Int. J. Mol. Sci..

[65] Corcoran B.M., Stanton C., Fitzgerald G., Ross R.P. (2008). Life under stress: The probiotic stress response and how it may be manipulated. Curr. Pharm. Des..

[66] Wesche A.M., Gurtler J.B., Marks B.P., Ryser E.T. (2009). Stress, sublethal injury, resuscitation, and virulence of bacterial food pathogens. J. Food Prot..

[67] Corcoran B.M., Stanton C., Fitzgerald G.F., Ross R.P. (2005). Survival of probiotic lactobacilli in acidic environments is enhanced in the presence of metabolizable sugars. Appl. Environ. Microbiol..

[68] Fortier L.C., Tourdot-Maréchal R., Diviès C., Lee B.H., Guzzo J. (2003). Induction of Oenococcus oeni H+-ATPase activity and mRNA transcription under acidic conditions. FEMS Microbiol. Lett..

[69] Verruck S., de Carvalho-Wolf M., Rodrigues L.G., Amante E.R., Werneck-Vieira C.R., de Mello-Castanho R.D.A., Schwinden-Prudencio E. (2017). Survival of *Bifidobacterium* BB-12 microencapsulated with full-fat goat’s milk and prebiotics when exposed to simulated gastrointestinal conditions and thermal treatments. Small Rumin. Res..

[70] Liu H., Gong J., Chabot D., Miller S.S., Cui S.W., Ma J., Zhong F., Wang Q. (2016). Incorporation of polysaccharides into sodium caseinate-low melting point fat microparticles improves probiotic bacterial survival during simulated gastrointestinal digestion and storage. Food Hydrocoll..

[71] Afzaal M., Khan A.U., Saeed F., Arshad M.S., Khan M.A., Saeed M., Maan A.A., Khan M.K., Ismail Z., Ahmed A. (2020). Survival and stability of free and encapsulated probiotic bacteria under simulated gastrointestinal conditions and in ice cream. Food Sci. Nutr..

[72] Krasaekoopt W., Watcharapoka S. (2014). Effect of addition of inulin and galactooligosaccharide on the survival of microencapsulated probiotics in alginate beads coated with chitosan in simulated digestive system, yogurt and fruit juice. LWT–Food Sci. Technol..

[73] Ozkan E.R., Demirci T., Ozturk H.I., Akin N. (2021). Screening *Lactobacillus* strains from artisanal Turkish goatskin casing Tulum cheeses produced by nomads via molecular and in vitro probiotic characteristics. J. Sci. Food Agric..

[74] Jensen H., Grimmer S., Naterstad K., Axelsson L. (2012). In vitro testing of commercial and potential probiotic lactic acid bacteria. Int. J. Food Microbiol..

[75] Lebeer S., Vanderleyden J., De Keersmaecker S.C. (2008). Genes and molecules of lactobacilli supporting probiotic action. Microbiol Mol. Biol. Rev..

[76] Ouwehand A.C., Salminen S. (2003). Invitro adhesion assays for probiotics and their invivo relevance: A review. Microb. Ecol..

[77] Ruas-Madiero P., Gueimonde M., Arigoni F., de los Reyes-Gavilan C.G., Margolles A. (2009). Bile Affects the Synthesis of Exopolysaccharides by *Bifidobacterium animalis*. Appl. Environ. Microb..

[78] Ruiz L., Sánchez B., Ruas-Madiedo P., Reyes-Gavilán C.G., Margolles A. (2007). Cell envelope changes in *Bifidobacterium animalis* ssp. lactis as a response to bile. FEMS Microbiol. Lett..

[79] Aboulfazli F., Baba A.S. (2015). Effect of Vegetable Milk on Survival of Probiotics in Fermented Ice Cream under Gastrointestinal Conditions. Food Sci. Technol. Res..

[80] Mishra V., Prasad D. (2005). Application of in vitro methods for selection of *Lactobacillus casei* strains as potential probiotics. Int. J. Food Microbiol..

[81] Furtado G.F., Almeida F.S., Sato A.C.K., Hubinger M.D. (2022). Model infant formulas: Influence of types of whey proteins and lipid composition on the in vitro static digestion behavior. Food Res. Inter..

[82] Aprikian O., Levrat-Verny M.A., Besson C., Busserolles J., Rémésy C., Demigné C. (2001). Apple favourably affects parameters of cholesterol metabolism and of anti-oxidative protection in cholesterol-fed rats. Food Chem..

[83] Munarin F., Tanzi M.C., Petrini P. (2012). Advances in biomedical applications of pectin gels. Int. J. Biol. Macromol..

[84] Shinohara K., Ohashi Y., Kawasumi K., Terada A., Fujisawa T. (2010). Effect of apple intake on fecal microbiota and metabolites in humans. Anaerobe.

[85] Krasaekoopt W., Bhandari B., Deeth H. (2004). The influence of coating materials on some properties of alginate beads and survivability of microencapsulated probiotic bacteria. Int. Dairy J..

[86] Li R., Zhang Y., Polk D.B., Tomasula P.M., Yan F., Liu L. (2016). Preserving viability of *Lactobacillus rhamnosus* GG in vitro and in vivo by a new encapsulation system. J. Control. Release.

[87] Pascale N., Gu F., Larsen N., Jespersen L., Respondek F. (2022). The Potential of Pectins to Modulate the Human Gut Microbiota Evaluated by In Vitro Fermentation: A Systematic Review. Nutrients.

[88] Gerez C.L., de Valdes G.F., Gigante M.L., Grosso C.R.F. (2012). Whey protein coating bead improves the survival of the probiotic *Lactobacillus rhamnosus* CRL 1505 to low pH. Lett. Appl. Microbiol..

[89] Gebara C., Chaves K.S., Ribeiro M.C.E., Souza F.N., Grosso C.R.F., Gigante M.L. (2013). Viability of *Lactobacillus acidophilus* La5 in pectin–whey protein microparticles during exposure to simulated gastrointestinal conditions. Food Res. Int..

[90] Kadlec R., Jakubec M. (2014). The effect of prebiotics on adherence of probiotics. J. Dairy Sci..

[91] Laparra J.M., Sanz Y. (2009). Comparison of in vitro models to study bacterial adhesion to the intestinal epithelium. Lett. Appl. Microbiol..

[92] Tuomola E.M., Salminen S.J. (1998). Adhesion of some probiotic and dairy *Lactobacillus* strains to Caco-2 cell cultures. Int. J. Food Microbiol..

[93] Lim S.M., Ahn D.H. (2012). Factors affecting adhesion of lactic acid bacteria to Caco-2 cells and inhibitory effect on infection of Salmonella typhimurium. J. Microbiol. Biotechnol..

[94] Collado M.C., Surono I., Meriluoto J., Salminen S. (2007). Indigenous dadih lactic acid bacteria: Cell-surface properties and interactions with pathogens. Food Microbiol. Safety.

[95] Nikolic M., Jovcic B., Kojic M., Topisirovic L. (2010). Surface properties of *Lactobacillus* and *Leuconostoc* isolates from homemade cheeses showing auto-aggregation ability. Eur. Food Res. Technol..

