# Supplementary material for: Sheep Milk Symbiotic Ice Cream: Effect of Inulin and Apple Fiber on the Survival of Five Probiotic Bacterial Strains during Simulated In Vitro Digestion Conditions
Source: Nutrients. 2022 Oct 23;14(21):4454. doi: 10.3390/nu14214454 (PMC9655080; doi:10.3390/nu14214454)
Supplement: Supplementary file 1 [file nutrients-14-04454-s001.zip › nutrients-1978759-supplementary.pdf]

Table S1. Analysis of variance (ANOVA) p-values on the effects of type of bacteria, inulin and fiber on viable counts of probiotic bacteria in ice cream before and during simulated *in vitro* digestion.

**Stage**	**Inulin p-Values**	**Apple fiber p-Values**	**Type of bacteria p-Values**	**Inulin × Apple fiber p-Values**	**Inulin × Type of bacteria p-Values**	**Type of bacteria × Apple fiber p-Values**	**Inulin × Apple fiber × Type of bacteria p-Values**
Before digestion	n.s. 0.9951	n.s. 0.7823	↑ 0.0000	n.s. 0.7770	↑ 0.0003	↑ 0.0001	n.s. 0.0612
Oral stage	n.s. 0.5761	n.s. 0.3482	↑ 0.0000	n.s. 0.4285	↑ 0.0001	↑ 0.0000	n.s. 0.7024
Stomach stage	↑ 0.0289	n.s. 0.8282	↑ 0.0000	n.s. 0.1280	↑ 0.0122	↑ 0.0487	↑ 0.0126
Small intestine	↑ 0.0173	↑ 0.0001	↑ 0.0000	↑ 0.0023	↑ 0.0000	↑ 0.0000	↑ 0.0001

* interaction ↑ indicates significant effect $p < 0.05$; n.s. - no significant effect.
